# Determination of superoxide dismutase mimetic activity in common culinary herbs

**DOI:** 10.1186/2193-1801-3-578

**Published:** 2014-10-01

**Authors:** Magali Chohan, Declan P Naughton, Elizabeth I Opara

**Affiliations:** School of Sport, Health and Applied Science, St Mary’s University, Waldegrave Road, Strawberry Hill, Twickenham, TW1 4SX UK; School of Life Sciences, Kingston University, Penrhyn Road, Kingston upon Thames, KT1 2EE UK

**Keywords:** Culinary herbs, Superoxide dismutase mimetic activity, Antioxidant, Anti-inflammatory

## Abstract

**Background:**

Under conditions of oxidative stress, the removal of superoxide, a free radical associated with chronic inflammation, is catalysed by superoxide dismutase (SOD). Thus in addition to acting as an antioxidant, SOD may also be utilized as an anti-inflammatory agent. Some plant derived foods have been shown to have SOD mimetic (SODm) activity however it is not known if this activity is possessed by culinary herbs which have previously been shown to possess both antioxidant and anti-inflammatory properties. The aim of the study was to ascertain if the culinary herbs rosemary, sage and thyme possess SODm activity, and to investigate the influence of cooking and digestion on this activity. Transition metal ion content was also determined to establish if it could likely contribute to any SODm activity detected.

**Findings:**

All extracts of uncooked (U), cooked (C) and cooked and digested (C&D) herbs were shown to possess SODm activity, which was significantly correlated with previously determined antioxidant and anti-inflammatory activities of these herbs. SODm activity was significantly increased following (C) and (C&D) for rosemary and sage only. The impact of (C) and (C&D) on the SODm for thyme may have been influenced by its transition metal ion content.

**Conclusions:**

SODm activity may contribute to the herbs’ antioxidant and anti-inflammatory activities however the source and significance of this activity need to be established.

## Background

The purported health promoting effects of culinary herbs may in part be due to their antioxidant and anti-inflammatory activities (Tapsell et al. 2006, Dhandapani et al. 2007, Lin and Lin 2008, Chohan et al. 2012, Baker et al. 2013). Culinary herbs are rich in polyphenols, which are the main contributors to their high antioxidant capacities (Zheng & Wang 2001, Dragland et al. 2003, Halvorsen et al. 2006, Chohan et al. 2012). With regards to their anti-inflammatory activity, due to the complexity of the inflammatory response it is likely that these herbs elicit their anti-inflammatory activity via a number of pathways and mediators. Recent studies support this theory as culinary herbs have been shown to inhibit a range of pro-inflammatory mediators (Tsai et al*.*^2007^, Chohan et al. 2012, Ho and Chang 2012, Kim et al. 2012, Baker et al. 2013).

The potent free radical superoxide (O_2_^•-^), is produced as part of the acute inflammatory response and is a known modulator of this response (Halliwell et al. 1988, Guzik et al. 2003, Yasui and Baba 2006). However, overproduction of superoxide has been associated with chronic inflammation, which can contribute to the development of a number of chronic diseases including cardiovascular disease and cancer (Yasui and Baba 2006). The enzyme superoxide dismutase (SOD) is one of the most important cellular antioxidant enzymes in biological systems. It catalyses the removal of superoxide by dismutation into hydrogen peroxide (H_2_O_2_) and oxygen, the H_2_O_2_ is then converted to water and oxygen by catalase. This action of SOD indicates that it may, in addition to being an antioxidant, be utilized as an anti-inflammatory agent (Yasui and Baba 2006). Studies have reported that plants and plant derived foods are able to either enhance or mimic SOD activity (Balaraman et al. 2004, Vouldoukis et al. 2004, Yoo et al. 2008, Thring et al. 2009, Candy et al. 2013, Carillon et al. 2013). Furthermore, this SOD mimetic (SODm) activity is associated with a decrease in pro-inflammatory mediators (Vouldoukis et al*.*^2004^, Carillon et al. 2013). Therefore, such activity may contribute not only to the antioxidant property of culinary herbs but also to their anti-inflammatory activity. Thus, the aim of this study was to establish if common culinary herbs possess SODm activity. Furthermore, to address the possible influence of preparation and consumption, the effect of cooking and digestion on this activity was also investigated.

## Materials and methods

### Reagents

All reagents were purchased from Sigma Aldrich, Poole, UK.

### Culinary herbs

Rosemary (*Rosmarinus officinalis*), sage (*Salvia officinalis*) and thyme (*Thymus vulgaris*) dried were purchased from Neal’s Yard Remedies, Richmond, Surrey, UK and stored as described in Chohan et al. (2012).

### Preparation of herb extracts

Extracts of uncooked (U), cooked (C), cooked and digested *in vitro* (C&D) and blank digests were prepared as previously described by the authors (Chohan et al. 2012).

### Preparation of standardised (STD) herb extracts

A standardised amount of 30 mg/mL (STD) was prepared so as to determine the effect of cooking and digestion on the SODm activity of the same gram (g) amount of herb. These extracts were prepared as previously described by the authors (Chohan et al. 2012).

### Identification of SODm activity

Herb extracts (U), (C), (C&D) and (STD, uncooked), and their respective blanks were assessed for SODm activity using the nitroblue tetrazolium (NBT) assay (Fisher et al. 2003).

### Quantification of Transition Metal Ions by Inductively Coupled Plasma-Atomic Emission Spectrometry (ICP-AES)

The content of redox-active transition metals of the herbs (U, C and C&D), and their respective blanks, was established, using ICP-AES spectrometry (ULTIMA 2C, Jobin Yvon, France) to determine if correlations exist with SODm activities.

### Expression of data and statistical analysis

Data are expressed as means ± SD unless otherwise stated. Statistical analysis was carried out using SPSS for Windows. Results of the NBT (SODm) assay are presented as a percentage inhibition of formazan production by herb extracts. ANOVA and the post hoc Tukey test were used to compare SOD mimetic activities expressed in % inhibition of formazan production for herbs (U), (C), (C&D) and STD. ANOVA and the post hoc Tukey test were used to compare the levels of metal ions (μg/g of herb) for (U), (C) and (C&D) extracts and blank digests (n = 2). The level of significance is p ≤ 0.05.

## Results

### SODm activity of herb extracts

All the herb extracts possessed SODm activity, which was not diminished by cooking and digestion. The SODm activities (Table 1) of rosemary and sage (U) were significantly lower than those of their (C) counterparts (p ≤ 0.05) and their (C&D) counterparts (p < 0.05). There was no significant difference between the SODm for thyme (U), (C) and (C&D) (p = 0.095). STD herbs possessed the highest SODm activities and all were significantly higher than those of their respective (U), (C) and (C&D) counterparts (p ≤ 0.05) except for sage (C&D) (p = 0.068). To gain some insight into the possible sources and significance of SODm activity, the SODm values for the prepared samples were compared with their respective total phenolic content (expressed as gallic acid equivalence (GAE)), antioxidant capacity (expressed as trolox equivalence antioxidant capacity) and anti-inflammatory activity values (expressed as inhibition of interleukin- 8 release from human peripheral blood lymphocytes), which were previously published by the authors (Chohan *et al.*^2012^) using Spearman rank correlation analysis. The analyses showed a significant association with GAE, TEAC, and % inhibition of IL-8 release: (r_*s*_ = 0.713, p ≤ 0.05 for SODm vs. estimated total phenolic content, r_*s*_ =0.699, p ≤ 0.05 for SODm vs. TEAC, r_*s*_ =0.713 p ≤ 0.05 for SODm vs. inhibition of IL-8 from PBLs exposed to TNF-α, and r_*s*_ =0.622 p ≤ 0.05 for SODm vs. inhibition of IL-8 from PBLs exposed to H_2_O_2_).

**Table 1:** **SOD mimetic activities and ICP-AES data for uncooked (U), cooked (C) and cooked and digested** ***in vitro*** **(C&D) herb extracts**

Sample	SODm % inhibition formazan production (n = 3) ^a^	Cu (μg/g) n = 2	Fe (μg/g) n = 2	Mn (μg/g) n = 2	Ni (μg/g) n = 2	V (μg/g) n = 2	Zn (μg/g) n = 2
Rosemary (U)	24.5 ± 1.8	0.69 ± 0.05	0.77 ± 0.06	2.96 ± 0.03	0.36 ± 0.08	1.13 ± 0.04	1.93 ± 0.01
Rosemary (C)	32.9 ± 1.4*	0.36 ± 0.01	0.57 ± 0.03	2.58 ± 0.03	0.31 ± 0.11	1.03 ± 0.04	1.71 ± 0.04
Rosemary (C&D)^**b**^	38.6 ± 3.9*	4.27 ± 0.80**	1.00 ± 0.61	10.39 ± 1.72**	0.56 ± 0.14	4.43 ± 0.74**	1.51 ± 0.91
Rosemary (STD)	65.5 ± 14.1	ND	ND	ND	ND	ND	ND
Sage (U)	19.5 ± 4.5	0.92 ± 0.03	1.33 ± 0.04	6.40 ± 0.01	0.11 ± 0.15	3.58 ± 0.01	7.11 ± 0.09
Sage (C)	27.3 ± 2.5*	1.29 ± 0.03*	1.22 ± 0.03	6.40 ± 0.33	0.15 ± 0.06	3.58 ± 0.12	8.22 ± 0.20*
Sage (C&D)^**b**^	32.8 ± 0.8*	2.36 ± 0.02**	5.61 ± 0.04**	12.32 ± 0.14**	0.01 ± 0.00	4.82 ± 2.10	7.29 ± 0.13
Sage (STD)	58.3 ± 18.6	ND	ND	ND	ND	ND	ND
Thyme (U)	36.9 ± 6.2^**NS**^	1.30 ± 0.02	2.02 ± 0.00	18.2 ± 0.04	0.27 ± 0.15	3.18 ± 0.00	6.88 ± 0.06
Thyme (C)	31.9 ± 1.5 ^**NS**^	1.86 ± 0.44	2.68 ± 0.46	24.4 ± 4.59	0.40 ± 0.14	3.82 ± 0.53	9.17 ± 1.72
Thyme (C&D)^**b**^	28.6 ± 1.7^**NS**^	3.09 ± 0.75	3.41 ± 0.66	26.28 ± 1.77	0.11 ± 0.04	6.78 ± 0.40**	8.29 ± 0.99
Thyme (STD)	70.8 ± 4.5	ND	ND	ND	ND	ND	ND

Herbs uncooked (U), cooked (C), cooked and digested *in vitro* (C&D) and standardised extracts (STD); ^a^SODm: Superoxide Dismutase mimetic activity; 33 units/mL of SOD was used as a positive control 87% ±3.7. ^b^Blank digests values were subtracted from herbs values (C&D). *significantly higher than (U) counterpart (p < 0.05), **significantly higher than both (U) and (C) counterparts (p < 0.05); ^NS^ no significant differences between thyme (U), (C) and (C&D) (p = 0.095). ND: not determined.

### ICP-AES analysis of herb extracts

Although overall the (C&D) extracts had the highest individual transition metal ion contents, there was no clear and consistent pattern. However, out of the three herbs, thyme, for each treatment (31.85 μg/g, 42.33 μg/g and 47.96 μg/g U, C and C&D respectively) had the highest total transition metal ions content based on the means of the constituent metal ions which was mainly due to their relatively high Mn content.

## Discussion

The aim of this study was to determine if herbs known to possess antioxidant capacity and anti-inflammatory activity that are influenced by preparatory and digestive processes (Chohan et al. 2012) possess SODm activity, and if so how this activity is affected by these processes.

Extracts of all the herbs (C&D) had higher amounts of Cu, Fe, Mn, and V than their (U) and (C) counterparts, (although this was not always significant), and this may be due to the acid digestion of the herbs at the simulated gastric fluid stage causing the release of these metal ions. Transition metal ions are known to enhance oxidative damage by Fenton and Haber-Weiss reactions (Fisher and Naughton 2005). Phenolic acids with catechol groups, which are the polyphenols that predominate in the herbs investigated, have been shown to chelate transition metal ions to form complexes and thus decrease oxidative damage to cell membranes (Psotova et al. 2003). However, in the presence of oxygen and transition metal ions, the redox cycling of these phenolic acids can lead to the formation of reactive oxygen species. Furthermore, the pro-oxidant/antioxidant activity of transition metal ion/phenolic acid complexes that form as a result of this redox cycling, depends on the metal reducing properties and chelating abilities of the phenolic acids (Sakihama et al. 2002). Chohan et al*.* (2012) reported that (C&D) increases, significantly, the total phenolic content of rosemary, sage and thyme. Thus, based on the mechanism described above, with this increase in polyphenol content, an increase in the chelation and/or reduction of the metal ions should result in a decrease in antioxidant activity for (C&D) herbs. However, the opposite occurred for the samples of rosemary (C&D) and sage (C&D) both in terms for their SODm activity (in the present study) and their antioxidant capacity (Chohan et al. 2012). It may be that the phenolic acids increased in insufficient quantities to reduce and/or chelate the transition metal ions and thus the balance was kept in favour of antioxidant activity. In the case of thyme (C&D), the balance in favour of antioxidant activity may have been compromised by pro-oxidant activity in the NBT assay, possibly generated by the higher levels of Mn and V, which may have occurred at the expense of SODm activity and thus may explain why this activity for thyme (C&D) was lower compared to rosemary and sage (C&D) and not higher compared to its (U) and (C) counterparts.

The correlation analysis clearly shows that there is a strong and significant association between SODm activity in the herb samples and their respective GAE, TEAC and anti-inflammatory activity. Based on this analysis it is tempting to speculate that the herbs’ polyphenols are sources of the SODm activity as suggested by Hunaefi and Smetanska (2013). This analysis also suggests that SODm activity contributes to the herbs’ anti-inflammatory activity which is supported by the literature for other foods (Vouldoukis et al. 2004, Carillon et al. 2013). SODm activities were also assessed as a function of redox-active transition metal ion content (Fisher et al. 2003, Fisher and Naughton 2005) however no significant relationship was found.

## Conclusions

In summary the current study shows that common culinary herbs possess SODm activity, which is not diminished by cooking and digestion. Correlation analysis suggests that this activity is associated with the herbs high polyphenol content, antioxidant capacity and anti-inflammatory action. Further work is required to fully determine its potential benefit *in vivo* as herbs are invariably consumed with a variety of foodstuffs and thus considerable scope exists for synergistic and antagonistic interactions with components released during digestion.
